# Dual Sinus Origins of the Left Coronary Artery From the Right and Left Coronary Sinus

**DOI:** 10.1016/j.cjco.2025.04.015

**Published:** 2025-04-27

**Authors:** Federico Oliveri, Martijn Van Oort, Frank Van der Kley, J. Wouter Jukema, José Montero-Cabezas

**Affiliations:** a Department of Cardiology, Leiden University Medical Center, Leiden, The Netherlands; b Netherlands Heart Institute, Utrecht, The Netherlands

A 51-year-old man was referred for evaluation of typical angina. After antianginal medications were given, coronary computed tomography (CT), was performed, which revealed significant stenosis in the distal right coronary artery (RCA) and circumflex artery (LCX), as well as an anomalous origin of the left coronary artery (LCA; Fig. 1). Specifically, the confluence of the mid-distal left anterior descending (LAD) artery and the proximal LCX was supplied by 2 distinct arteries: (1) a small, separate branch originating from the left coronary sinus (native LCA); and (2) an anomalous LCA of the right sinus (ALCA-R), arising from a shared origin with the RCA in the right sinus, which coursed anteriorly and intramyocardially through the septum to the distal LAD territory (see Videos 1  and 2  view videos online.).^1^ The invasive procedure confirmed the CT findings (see Videos 3  and 4  view videos online). Competitive flow from the ALCA-R and the native LCA was noted. Fractional flow reserve was performed to assess an intermediate stenosis in the mid-LAD artery, yielding a negative result (fractional flow reserve = 0.86). The case was discussed in a congenital heart team meeting, and the CT findings were reviewed with an experienced radiologist. The consensus decision was to proceed with percutaneous coronary intervention (PCI) for the distal RCA and LCX (see Video 5  view video online). PCI of the LCX was performed retrogradely via the ALCA-R using a catheter extension, because the native LCA from the left coronary sinus presented severe tortuosity (Fig. 1F).

**Figure 1:**
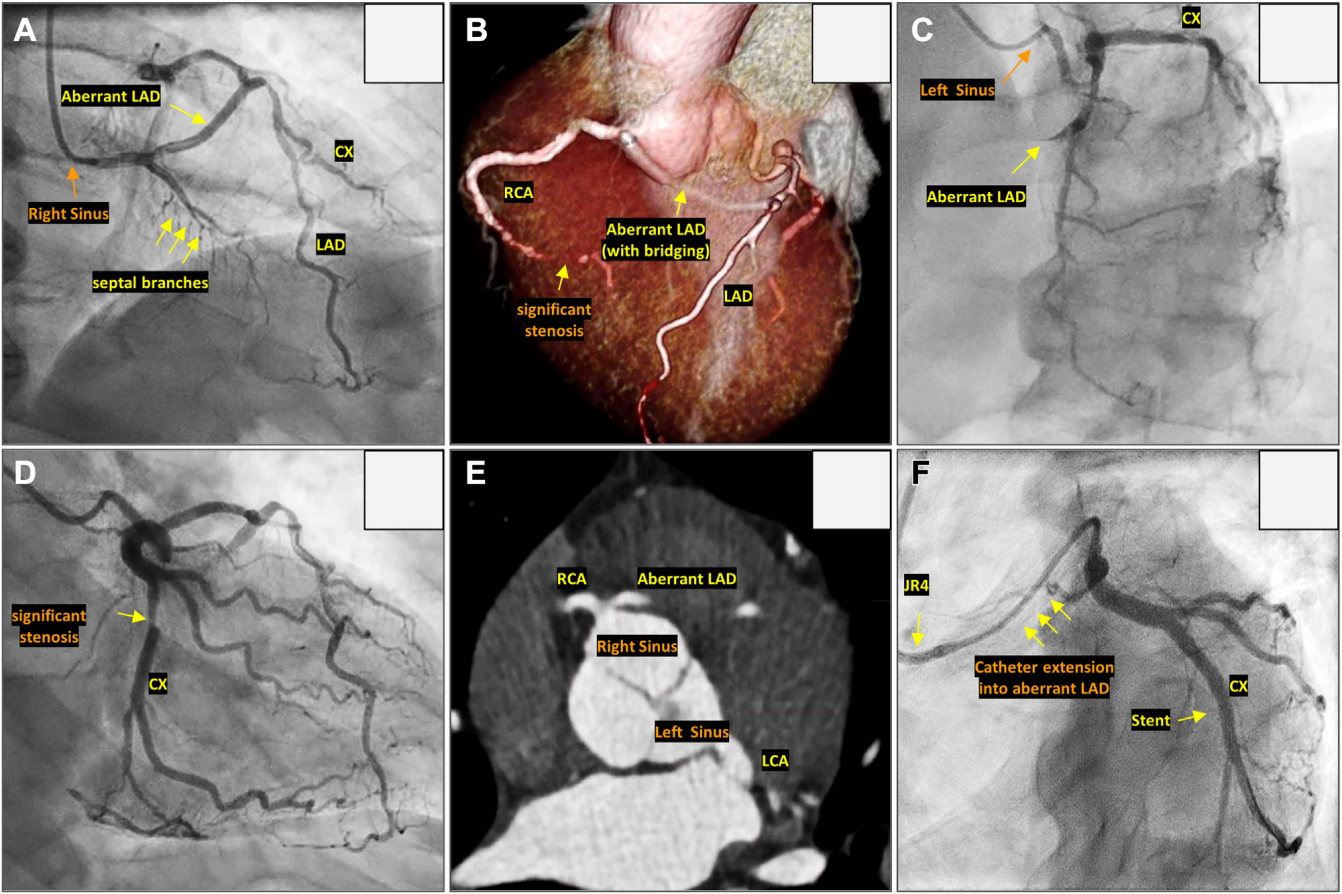
(**A**) Coronary angiography showing an aberrant left coronary artery (LCA) arising from the right coronary sinus; (**B**) 3D coronary computed tomography scan showing the aberrant LCA originating from the right coronary sinus with an intramyocardial course; (**C**) coronary angiography from the left coronary sinus revealing competing flow between the aberrant and native LCA toward the mid-distal left anterior descending (LAD) artery; (**D**) coronary angiography showing significant mid-circumflex artery (CX) stenosis; (**E**) computed tomography scan showing the dual origin of the LCA; and (**F**) final result of percutaneous coronary intervention of the CX. JR4, Judkins right 4; RCA, right coronary artery.

To the best of our knowledge, this is the first reported case of an anomalous origin of the LCA from the right sinus coupled with another branch arising from the left sinus. Although an ALCA-R is associated with an elevated risk of sudden cardiac death,^2^ the presence of 2 distinct origins might provide a degree of protection against ischemia, as long as no significant epicardial stenosis is present. This case also highlights the value of multidisciplinary discussion in revascularization management and the feasibility of successful PCI in the context of complex coronary anatomy.

**Novel Teaching Points**

• To our knowledge, this is the first reported case of an anomalous origin of the LCA from the right sinus, coupled with another branch arising from the left sinus.
• Multidisciplinary evaluation to optimize the revascularization strategy is pivotal.
• PCI of the LCX from the ALCA-R is feasible.

## Supplementary Material

Video 1 Computed tomography scan showing the dual origin of the left coronary artery from the right and left coronary sinuses.

Video 2 Computed tomography scan showing the course of the aberrant left anterior descending artery.

Video 3 Coronary angiography illustrating the course of the aberrant left anterior descending artery.

Video 4 Coronary angiography showing the competitive flow between the native left coronary artery and the aberrant left anterior descending artery.

Video 5 Percutaneous coronary intervention of the circumflex artery.
